# Superior cardiometabolic and cellular adaptive responses to multiple *versus* single daily sessions of high-intensity interval training in Wistar rats

**DOI:** 10.1038/s41598-022-24906-y

**Published:** 2022-12-07

**Authors:** Bruno Ferreira Mendes, Liliane Vanessa Costa-Pereira, Júllia Alves de Andrade, Caíque Olegário Diniz Magalhães, Ramona Ramalho Souza de Pereira, Elizabethe Adriana Esteves, Ricardo Cardoso Cassilhas, Eric Francelino Andrade, Fernando Gripp, Flávio Castro de Magalhães, Kinulpe Honorato Sampaio, Fabiano Trigueiro Amorim, Marco Fabrício Dias-Peixoto

**Affiliations:** 1 Multicenter Graduate Program in Physiological Sciences, Federal University of the Jequitinhonha and Mucuri Valleys, Diamantina, MG Brazil; 2 Graduate Program in Health Sciences, Federal University of the Jequitinhonha and Mucuri Valleys, Diamantina, MG Brazil; 3 Laboratório Experimental de Treinamewnto Físico (LETFis), Department of Physical Education, Federal University of the Jequitinhonha and Mucuri Valleys, Diamantina, MG Brazil; 4 Department of Health, Exercise, and Sports Sciences, University of New Mexico, Albuquerque, NM 87131-0001 USA

**Keywords:** Risk factors, Mitochondria

## Abstract

This study aimed to compare in rats the cardiometabolic and cellular adaptative responses to 8 weeks of high-intensity interval training (HIIT) performed in a single (1xHIIT) or three shorter daily sessions (3xHIIT). Male Wistar rats were assigned to untrained (n = 10), 1xHIIT (n = 10), and 3xHIIT (n = 10) groups. Both HIIT groups performed 15 min of a treadmill run five times per week for 8 weeks. The 1xHIIT performed single daily sessions of 15 min, and the 3xHIIT performed three daily sessions of 5 min with an interval of 4 h between sessions. Resting VO_2_ and VO_2_max were measured using a metabolic chamber; blood pressure and heart rate were measured by plethysmography; body composition was estimated by DEXA; Glucose and insulin tolerance tests were performed; after euthanasia, hearts, gastrocnemius, and visceral fat were harvested for analysis of cardiac function, histology, and morphology. Mitochondrial densities of the gastrocnemius and left ventricle muscles were determined by electron microscopy. 3xHIIT induced similar positive adaptative responses to 1xHIIT on resting VO_2_ and VO_2_max, cardiac function, and mitochondria density. 3xHIIT was superior to 1xHIIT in reducing visceral fat weight and adipocyte size and improving insulin tolerance. Multiple short daily bouts of HIIT may be superior to single HIIT daily sessions in improving cardiometabolic and cellular adaptations in rats.

## Introduction

In recent decades, robust scientific evidence has shown the association between sedentary behavior and cardiometabolic disease risk^1^. Before the Coronavirus disease 2019 pandemic (COVID-19), data from 168 countries (representing 96% of the world's population), including 1.9 million people, indicated that 27.5% of adults were insufficiently physically active^2^, and the long-term confinements used to prevent the spreading of COVID-19 have increased cardiometabolic diseases related to sedentary behavior worldwide^3^. Thus, physical exercise programs that can be performed time-efficiently have been an important recommendation to maximize engagement and enjoyment during confinement periods^4^.

Breaking up sedentary behavior with brief bouts of vigorous exercise throughout the day has been proposed as a feasible and efficient approach to improving cardiometabolic health^5,6^. Using the term “exercise snacks”, Francois et al.^7^ demonstrated that brief, intense exercise bouts before main meals improved glycemic control in individuals with insulin resistance. Recently, Little et al.^8^ reported that 6 weeks of vigorous exercise training performed in a single daily session (3 × 20-s ‘all-out’ cycling bouts interspersed with 3-min rest) or accumulated in 3 shorter sessions over the course of a day (3 × 20-s ‘all-out’ cycling bouts separated by 1–4-h rest) induced similar increases in pea6k oxygen consumption (VO2peak) in young sedentary adults. Although other recent studies have also shown that “exercise snacks” improved some markers of cardiometabolic health^9,10^, it is still unknown whether this approach is as effective as single daily exercise sessions in improving cardiometabolic function and cellular adaptations. We recently showed that 8 weeks of a Moderate Intensity-Continuous Training (MICT) protocol (60–75% maximal workload) performed in 3 short daily sessions (10–20 min per session) was superior to single daily sessions (30–60 min) in reducing visceral fat weight and adipocyte size in young Wistar rats. Of note, these results occurred independently of total energy intake^11^.

However, the study from Costa Pereira et al. had some limitations. First, MICT may induce inferior training adaptations than other time-efficient exercise protocols, such as HIIT protocols^5^; second, Costa-Pereira et al. used a swimming protocol, which is not as usual as other exercise protocols, e.g., running protocols; and finally, Some critical analyses of classical cellular adaptations to training, such as skeletal and cardiac muscle mitochondrial densities, were not performed.

To our knowledge, no previous studies have investigated cardiometabolic and cellular responses to a HIIT protocol performed with single *versus* multiple daily sessions. Therefore, in the present study, we investigated in young Wistar rats the effects of 8 weeks of HIIT performed in a single daily session (1 daily session of 15 min) versus multiple daily sessions (3 daily sessions of 5 min with an interval of 4 h between sessions) on cardiac function and hypertrophy, visceral fat weight and adipocyte size, glucose tolerance and insulin sensitivity, and mitochondrial densities of the gastrocnemius and left ventricle muscles. We hypothesized that HIIT performed with multiple daily sessions would have similar or superior beneficial effects to HIIT performed with single daily sessions on markers of cardiometabolic health of young Wistar rats.

## Materials and methods

### Animals and study design

Experimental protocols were performed following the Guide for the Care and Use of Laboratory Animals published by the National Institute of Health (NIH Publication, 1996) and approved by the local ethical committee on Animal Use from the Federal University of Jequitinhonha and Mucuri Valleys (protocol #031/2016).

**Figure 1:**
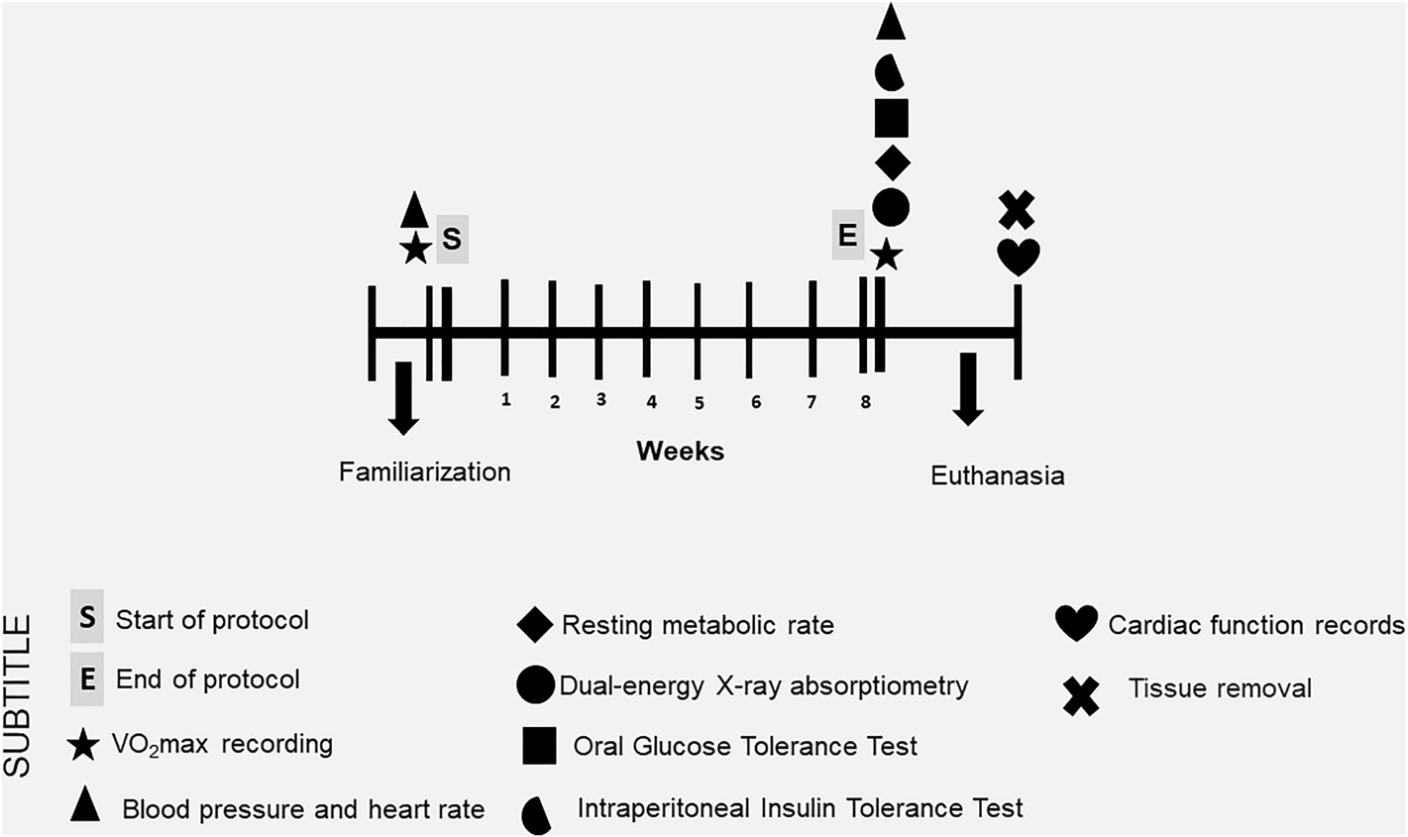
Experimental design.

Sixty-day-old male Wistar rats weighing 220 to 260 g were obtained from the animal facility of the Biological Science Institute of the Federal University of Minas Gerais (Brazil). The animals were housed in individual cages in a controlled environment (22–23 °C, 50% humidity, and low noise) and maintained on a 12:12 h light–dark cycle (lights turn on at 6 p.m. and turn off at 6 a.m.) with free access to standard laboratory food pellets (Nuvilab Nutrients LTDA, Colombo, PR, Brazil) and water. All experiments were performed on the dark cycle. Thirty rats were randomly assigned to Untrained (n = 10), HIIT performed with single daily sessions (1xHIIT, n = 10), and HIIT performed with three shorter daily sessions (3xHIIT, n = 10) groups. The exercise training protocol was performed for 8 weeks. Body weight and food intake were recorded weekly. Maximal oxygen consumption (VO_2_max), blood pressure, and heart rate were recorded before and after the training period. Body composition, resting oxygen consumption, and glucose homeostasis were evaluated after the training period. Thereafter, animals were euthanized, and hearts, gastrocnemius muscles, and visceral fat were harvested for cardiac function experiments and morpho-histological analyses. Figure 1 illustrates the experimental design.

### Pre-training measurements

#### Blood pressure and heart rate

The rats were previously familiarized with the noninvasive procedures for tail-cuff plethysmography for 5 days (MLT1020PPG IR Plethysmograph; ADInstruments, New South Wales, Australia). The tail systolic blood pressure and heart rate were recorded 48 h before and 48 h after the exercise training protocol as described by Costa Pereira et al.^11^.

#### *VO*_*2*_*max test*

The VO_2_max test was performed according to Hoydal et al.^12^. Briefly, the animals were familiarized with the treadmill using a low running velocity (10 m/min) for 10 min for five consecutive days. All animals performed the VO_2_max test at least 48 h after the last familiarization session with the treadmill. The VO_2_max test was performed at least 48 h before the first training session, and at least 48 after the last training session using a metabolic analyzer (Oxyleptro, Harvard Apparatus, Spain) coupled to a metabolic chamber (airflow = 1.0 L/min), which accommodated the rats. The calorimetric parameters were measured using a respiratory-based software program (software Metaoxy, Harvard Apparatus, Spain)^12^. After 10 min of warming up at 0.05 m/s, the treadmill running velocity was increased every 2 min by 3 m/min until the VO_2_max velocity [established when oxygen consumption (VO_2_) reaches a plateau despite running speed increase]. The treadmill speed at VO_2_max was considered the maximal speed (Vmax) and used for the exercise training prescription.

### Physical training protocol

**Figure 2:**
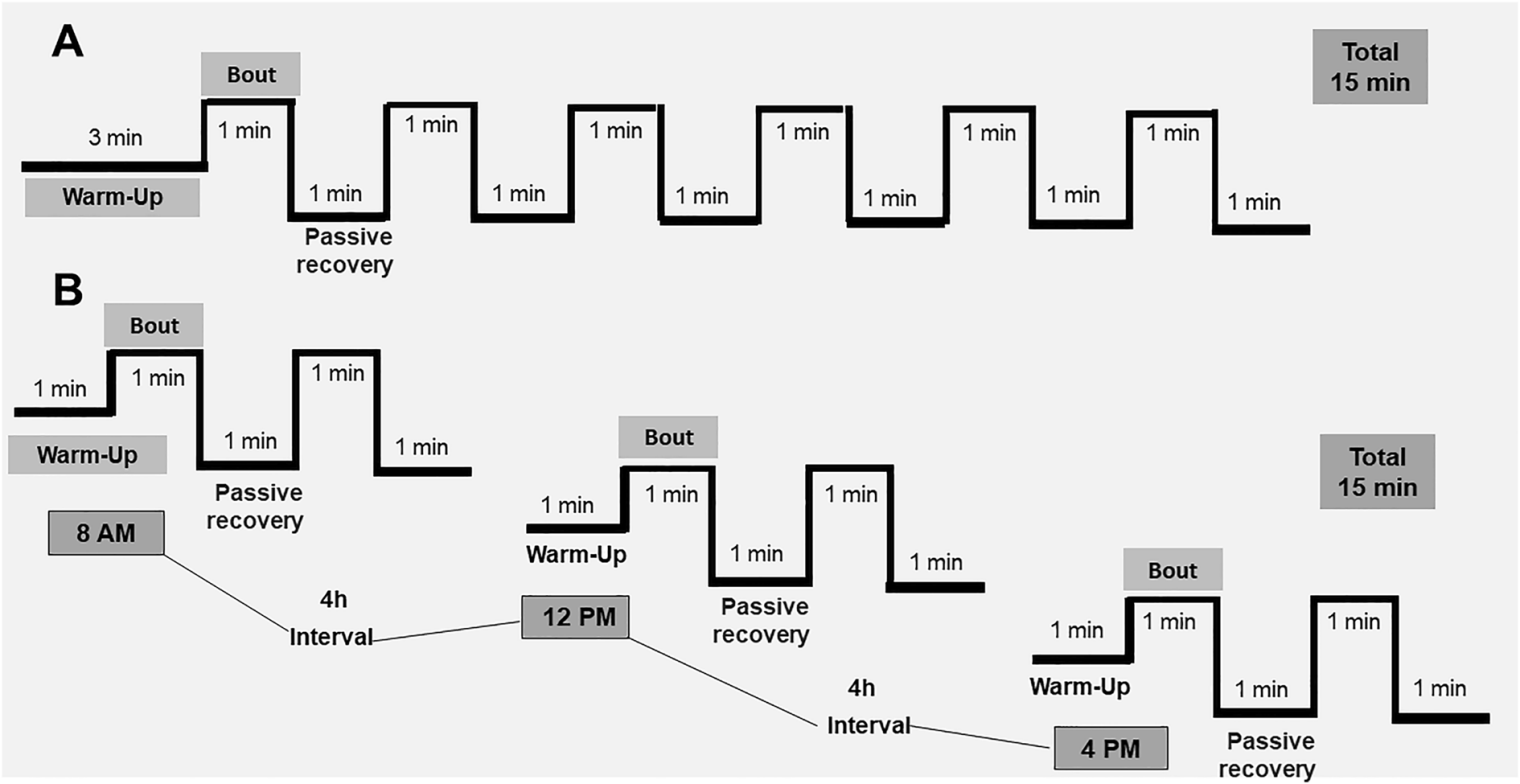
Physical training protocol. 1xHIIT: High-Intensity Interval Training performed in single daily sessions (**A**); 3xHIIT: High-Intensity Interval Training performed in three shorter daily sessions (**B**). Both training groups (1xHIIT and 3xHIIT) performed the HIIT protocols 5 days per week over 8 weeks. The 1 × HIIT group performed single daily sessions of 3 min of warm-up, followed by six bouts of 1 min interspersed by 1 min of passive recovery (sessions of 15 min daily, in total). The 3xHIIT Group performed three shorter daily sessions composed of 1-min warm-up, followed by two bouts of 1 min interspersed by 1 min of passive recovery, with an interval of 4 h between sessions (sessions of 15 min daily, in total).

At least 48 h after the VO_2_max test, the animals started the exercise training protocols (Fig. 2). The training was performed for 1xHIIT and 3xHIIT groups 5 days per week over 8 weeks. The 1xHIIT performed single daily sessions composed of 3 min of warm-up, followed by six bouts of 1 min interspersed by 1 min of passive recovery (sessions of 15 min daily, in total; Fig. 2A). The 3xHIIT performed three shorter daily sessions composed of 1 min of warm-up, followed by two bouts of 1 min interspersed by 1 min of passive recovery (total of 5 min), with an interval of 4 h between sessions (sessions of 15 min daily, in total; Fig. 2B). The training progression was based on the exercise intensity increasing without changing exercise volume for both groups: 1st week—6 × 1 min 85% Vmax; 2nd and 3rd weeks—90% Vmax; 4th and 5th weeks—95% Vmax; 6th, 7th, and 8th weeks—100% Vmax.

At the end of the 4th week, a second VO_2_max test was performed to establish a new maximal running speed (Vmax) and used for the subsequent exercise training sessions. For the 3xHIIT group, training sessions were performed at 8:00 a.m., 12:00 p.m., and 4:00 p.m. The 1xHIIT was split into three subgroups starting the training sessions at the same times as the 3-HIIT (8:00 a.m., 12:00 p.m., and 4:00 p.m.) to eliminate any possible circadian effect on training adaptations. The untrained group was placed in the same room where the HIIT groups performed the training protocol. Also, the untrained group exercised on the treadmill for 10 min at a speed of 10 m/min once a week. At the end of the 8th week, the VO_2_max test was repeated to measure the effectiveness of the exercise training.

### Post-training measurements and analyses

#### Dual-energy X-ray absorptiometry

Body composition was determined 24 h after the last exercise session by dual-energy X-ray absorptiometry (DEXA, LUntrainedar iDXA, G.E. Healthcare, USA). The animals received an intraperitoneal injection of ketamine (60 to 80 mg/kg) and xylazine (8 to 15 mg/kg) and were placed in the equipment scanning area. Then, the researcher adjusted the animal's position, ensuring that the demarcated sagittal line passed under the center of anatomical points such as the skull, spine, pelvis, and hind legs. Lean mass and fat body fat percentage were determined using a commercial software for animal analyses (enCORE, G.E. Healthcare, USA)^13^.

#### Resting oxygen consumption

Resting oxygen consumption was measured 72 h after the last exercise session and after overnight fasting (8 h) for 60 min. The rats were accommodated in a metabolic chamber (airflow = 1.0 L/min) coupled to a computer-monitored indirect calorimeter (Oxyleptro, Harvard Apparatus, Spain) for resting oxygen consumption records. The calorimetric parameters were measured using a respiratory-based software program (software Metaoxy, Harvard Apparatus, Spain)^14^.

#### Oral glucose tolerance test (OGTT)

OGTT was performed immediately after the resting oxygen consumption measurements. Dextrose was administered by gavage (2 g/kg of body weight at 50% solution). Blood glucose levels were determined by a small puncture of the rat tail immediately before (0 min) and at 30, 60, and 120 min after the gavage. Blood glucose levels were determined using an ACCU-CHEK (Advantage Glucose Analyzer, Roche Diagnostics Corporation, Indianapolis, IN, USA)^15^.

#### Intraperitoneal insulin tolerance test (IpITT)

Forty-eight hours after the OGTT, the animals fasted for eight hours, and then an intraperitoneal injection of insulin (1 I.U./kg BW) was administered. Blood glucose levels were determined following the same protocol of OGTT^15^. All data were normalized and presented as a percentage of baseline glucose concentration to eliminate potential differences in baseline glucose values among groups. The acquired glucose values were used to calculate the Area Under the Curve (AUC) (% baseline glucose × 60 min).

#### Euthanasia

After an intraperitoneal injection of 400 IU heparin, the animals were decapitated, and heart, gastrocnemius muscle, and visceral fat were harvested and weighed. Hearts were perfused (Langendorff technique) for cardiac function records. A fragment of the gastrocnemius muscle was processed (transmission electron microscopy) for mitochondrial density analyses, and portions of the retroperitoneal fat were removed, weighed, and fixed in 4% Bouin to measure adipocyte area^14–16^.

#### Cardiac function

The cardiac function was determined by the Langendorff perfusion system (ML785B2, ADInstruments). The thorax was opened, and the heart was dissected and retrogradely perfused in the Langendorff apparatus with Krebs–Ringer solution (in mmol⋅L − 1 : NaCl, 118.40; KCl, 4.70; KH_2_PO_4_, 1.17; MgSO_4_, 1.17; CaCl_2_, 2.50; glucose, 11.65; and NaHCO_3_, 26.30) at 37 ± 1 °C and constant pressure (65 mm Hg) and oxygenation (5% CO_2_ and 95% O_2_). Contractility (+ dP/dt), relaxation (− dP/dt) indexes and heart rate (HR) were calculated using the AcqKnowledge software (TSD 104A, Biopac Systems Inc., Santa Barbara, California, USA)^14^. After cardiac function records, left ventricles were dissected, weighed, divided into fragments, and analyzed by transmission electron microscopy for mitochondrial density quantification^16^.

#### Histological analysis

The fragments of retroperitoneal fat and gastrocnemius tissues were fixed in formalin (10%) for 48 h and subsequently submitted to dehydration with increasing alcohol gradient (70, 80, 90, and 100%) followed by infiltration and inclusion in paraffin. After assembly of the blocks, the samples were cut in a microtome in sections of 5 mm with intervals of 20 cuts. The sections were stained with hematoxylin and eosin for analysis. Cross-sections were used (~ 10 fields per animal), and the cell area length of ≥ 100 adipocytes or myocytes per animal, respectively, were evaluated^14^.

#### Mitochondrial density and morphology

Posterior mid-belly fragments of the gastrocnemius and left ventricle muscles were dissected from three animals per group. The specimens were fixed in Karnovsky’s solution (2.5% glutaraldehyde and 2% paraformaldehyde) in 0.1 M cacodylate buffer pH 7.4 overnight at 4˚C. They were post-fixed in a mixture of 2% (w/v) osmium tetroxide and 1.5% (w/v) potassium ferrocyanide for 2 h to enhance the contrast of organelles. Specimens were washed in distilled water and kept in 2% uranyl acetate overnight. Then, the samples were serially dehydrated in graded ethanol baths and embedded in Epon 812. Specimens were sectioned in 50 nm sections and stained with Reynolds lead citrate. Transmission electron microscopy (TEM) was performed using a FEI Tecnai G2—12 Spirit at 80 kV, exhibiting a point resolution of 0.49 nm. TEM was equipped with a SISMegaView 3 CCD camera, and acquired images showed 1373 × 1070 pixels. Twenty-five electron micrographs per animal were taken at a × 11,000 magnification. Images were randomly selected from central parts of muscle fibers and were analyzed with ImageJ. Volume densities (Vv) of normal and altered mitochondria were determined with the classic point method using a 130-point-grid (700 × 700 nm grid) projected onto each image. Altered mitochondria were defined as those that showed a swollen appearance with a rarefied matrix and damaged cristae, as previously described^17,18^.

### Statistical analysis

All data are expressed as mean ± standard deviation. The normality of data was checked using the Shapiro–Wilk test. All analyses were blinded, and data were analyzed using one (main factor = 3 groups) or two-way (3 groups vs. pre and post-training) analysis of variance followed by Tukey’s post-hoc test, where appropriate (Statistica software, v8.0, StatSoft, Inc). Statistical significance was set at *p* ≤ 0.05.

### Ethics approval

The study is reported in accordance with ARRIVE guidelines (https://arriveguidelines.org).

## Results

Table 1 presents the effects of 1xHIIT and 3xHIIT on VO_2_max, body mass, food intake, and blood pressure. These variables were similar for all groups at baseline. After the training period, only the trained groups presented an increase in VO_2_max (1xHIIT: *p* < 0.0001 and 3xHIIT: *p* < 0.0001), confirming the effectiveness of the training protocols. All groups showed an increase in body weight after the training period (Untrained: *p* < 0.0001; 1xHIIT: *p* < 0.0001; 3xHIIT: *p* < 0.0001). However, body weight was lower in both HIIT groups, compared with the Untrained group after the training period (1xHIIT vs. Untrained: *p* = 0.0027; 3xHIIT vs. Untrained: *p* = 0.0003; 1xHIIT vs. 3xHIIT: *p* = 0.9848). Food intake was similar among the groups during all experimental protocol period (1xHIIT vs. Untrained: *p* = 0.9976; 3xHIIT vs. Untrained: *p* = 0.9976; 1xHIIT vs. 3xHIIT: *p* > 0.9999). Compared with the baseline period, only the Untrained group presented an increase in blood pressure after 8 weeks of exercise training (Untrained: *p* < 0.0001; 1xHIIT: *p* = 0.8630; 3xHIIT: *p* = 0.6110). Moreover, 1xHIIT and 3xHIIT groups had lower blood pressure than Untrained animals after the training period (1xHIIT vs. Untrained: *p* = 0.0049; 3xHIIT vs. Untrained: *p* = 0.0049; 1xHIIT vs. 3xHIIT: *p* = 0.0393). Heart rate was similar for all groups before and after the training period (1xHIIT vs. Untrained: *p* = 0.8921; 3xHIIT vs. Untrained: *p* = 0.9998; 1xHIIT vs. 3xHIIT: *p* = 0.9748).

**Table 1:** Effects of HIIT performed in single versus three shorter daily sessions on VO_2_max, body weight, food intake, blood pressure, and heart rate.

	Untrained		1xHIIT		3xHIIT	
	Pre	Post	Pre	Post	Pre	Post
VO_2_max (mL kg^−1^ min^−1^)	52.2 ± 6.5	49.7 ± 3.1	49.45 ± 4.3	67.74 ± 5.1*^#^	51.57 ± 4.8	67.22 ± 3.0*^#^
Body mass (g)	242.8 ± 19.8	384.4 ± 2.1*	255.9 ± 14.7	349.8 ± 16.8*^#^	248.8 ± 14.9	344.0 ± 25.2*^#^
Food intake (g/wk)	118.3 ± 9.9	124.3 ± 3.7	116.5 ± 3.8	122.1 ± 8.6	119 ± 9.0	122.1 ± 5.0
Blood pressure (mmHg)	115.4 ± 4.2	136.5 ± 5.9*	120.8 ± 6.5	123.8 ± 9.17^#^	117.9 ± 10.1	124.3 ± 2.4^#^
Heart rate (bpm)	400.1 ± 30.7	378.3 ± 11.6	389.6 ± 36.3	365.3 ± 15.7	375.2 ± 18.1	374.8 ± 14.7

Data are presented as mean ± SD. N = 10/group. Two-way ANOVA followed by Tukey test. N = 10/group.

Untrained, Non-exercised group; 1xHIIT, High-intensity interval training performed in single daily sessions; 3xHIIT, High-intensity interval training performed in three shorter daily sessions.

*p < 0.05 PRE versus POST.

^#^*p* < 0.05 1xHIIT and 3xHIIT versus Untrained at post-training period.

**Figure 3:**
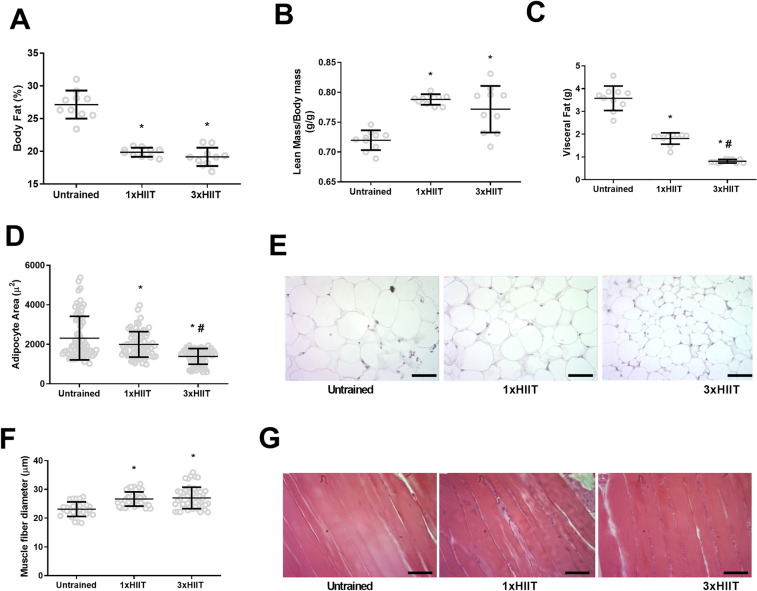
Effects of HIIT performed in single versus three shorter daily sessions on body composition, visceral fat mass, adipocyte size, and muscle fiber diameter (gastrocnemius muscle). Body fat percentage (%) (**A**, N = 10); lean mass (g/g) (**B**, N = 10); visceral fat mass (g) (**C**, N = 10); adipocyte area (µ^2^) **(D**, N = 72 cells/group from four independent experiments); representative H&E slides of adipocytes in the retroperitoneal fat (**E**); muscle fiber diameter (gastrocnemius, **F**, N = 49 fibers/group from four independent experiments); representative H&E slides of gastrocnemius muscle (**G**). Data are presented as mean ± SD. One-way ANOVA followed by Tukey test. *p < 0.05, HIIT groups versus Untrained group; and ^#^*p* < 0.05, 3xHIIT versus 1xHIIT group. 1xHIIT: High-Intensity Interval Training performed in single daily sessions; 3xHIIT: High-Intensity Interval Training performed in three shorter daily sessions.

Figure 3 presents the results of body composition (3A and 3B), visceral fat mass (3C), adipocyte size (3D and E), and muscle fiber diameter (3F and 3G) after training. Compared with the Untrained group, both HIIT groups presented a lower body fat percentage (1xHIIT vs. Untrained: *p* < 0.0001; 3xHIIT vs. Untrained: *p* < 0.0001; 1xHIIT vs. 3xHIIT: *p* = 0.5616; Fig. 3A) and higher lean mass (1xHIIT vs. Untrained: *p* < 0.0001; 3xHIIT vs. Untrained: *p* = 0.0002; 1xHIIT vs. 3xHIIT: *p* = 0.3295; Fig. 3B) with no differences between the trained groups. Both HIIT groups had lower visceral fat mass than the Untrained group, and, of note, the visceral fat mass of the 3xHIIT group was significantly lower than the 1xHIIT group (1xHIIT vs. Untrained: *p* < 0.0001; 3xHIIT vs. Untrained: *p* < 0.0001; 3xHIIT vs. 1xHIIT: *p* < 0.0001; Fig. 3C). Similar results were found for adipocyte area. The 3xHIIT group had the lowest adipocyte area among the groups (3xHIIT vs. 1xHIIT: *p* < 0.0001; 3xHIIT vs. Untrained: *p* < 0.0001) and the 1xHIIT rats had lower adipocyte area than the Untrained group (1xHIIT vs. Untrained: *p* = 0.0388; Fig. 3D,E). Both HIIT protocols induced similar skeletal muscle hypertrophy (1xHIIT vs. Untrained: *p* < 0.0001; 3xHIIT vs. Untrained: *p* < 0.0001; 1xHIIT vs. 3xHIIT: *p* = 0.8226; Fig. 3F,G).

**Figure 4:**
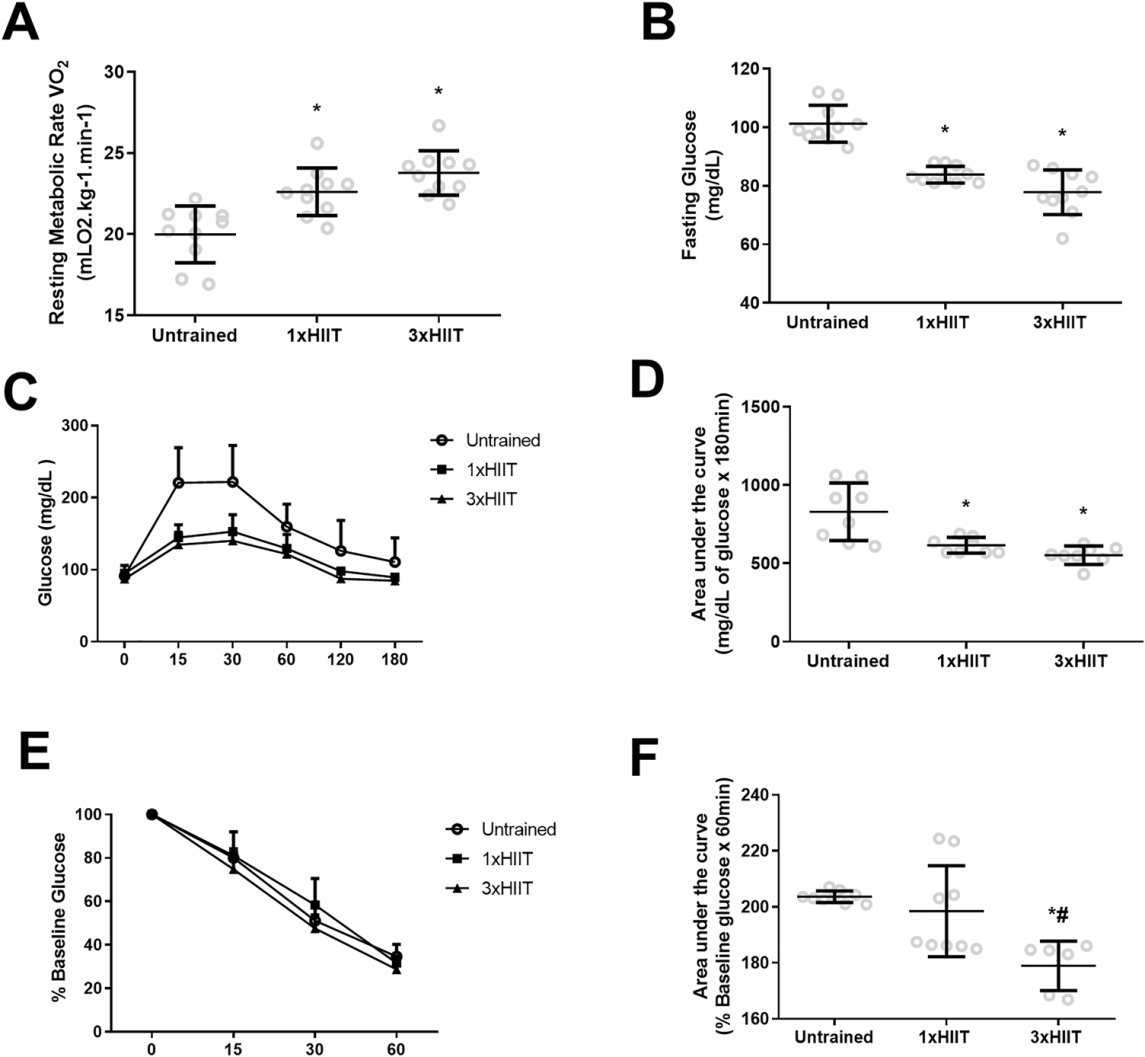
Effects of HIIT performed in single versus three shorter daily sessions on metabolic parameters. Resting metabolic rate (mL O_2_ Kg^−1^ min^−1^). (**A**) Resting glucose (mg/dL) (**B**); plasma glucose levels during oral glucose tolerance test (mg/dL), (**C**, **D**) after oral glucose challenge (2 g/Kg BW); plasma glucose levels (mg/dL) during insulin tolerance test (**E**, **F**) after i.p. insulin challenge (1U/kg BW). N = 10/group. Data are presented as mean ± SD. One or two-way ANOVA followed by the Tukey test. *p < 0.05, HIIT groups versus Untrained group; and ^#^*p* < 0.05, 3xHIIT versus 1xHIIT. 1xHIIT: High-Intensity Interval Training performed in single daily sessions; 3xHIIT: High-Intensity Interval Training performed in three shorter daily sessions.

Figure 4 presents the resting oxygen consumption and glucose metabolism results after training. Compared with the Untrained group, both trained groups showed similarly higher resting oxygen consumption (1xHIIT vs. Untrained: *p* = 0.0021; 3xHIIT vs. Untrained: *p* < 0.0001; 1xHIIT vs. 3xHIIT: *p* = 0.2258; Fig. 4A), lower fasting blood glucose levels (1xHIIT vs. Untrained: *p* < 0.0001; 3xHIIT vs. Untrained: *p* = 0.0001; 1xHIIT vs. 3xHIIT: *p* = 0.0801; Fig. 4B) and improved oral glucose tolerance (1xHIIT vs. Untrained: *p* = 0.0036; 3xHIIT vs. Untrained: *p* = 0.0003; 1xHIIT vs. 3xHIIT: *p* = 0.5182; Fig. 4C,D). Of note, only the 3xHIIT group showed improved insulin resistance during the intraperitoneal insulin tolerance test compared with the Untrained and 1xHIIT groups (1xHIIT vs. Untrained: *p* = 0.5898; 3xHIIT vs. Untrained: *p* = 0.0010; 3xHIIT vs. 1xHIIT: *p* = 0.0077; Fig. 4E,F).

**Figure 5:**
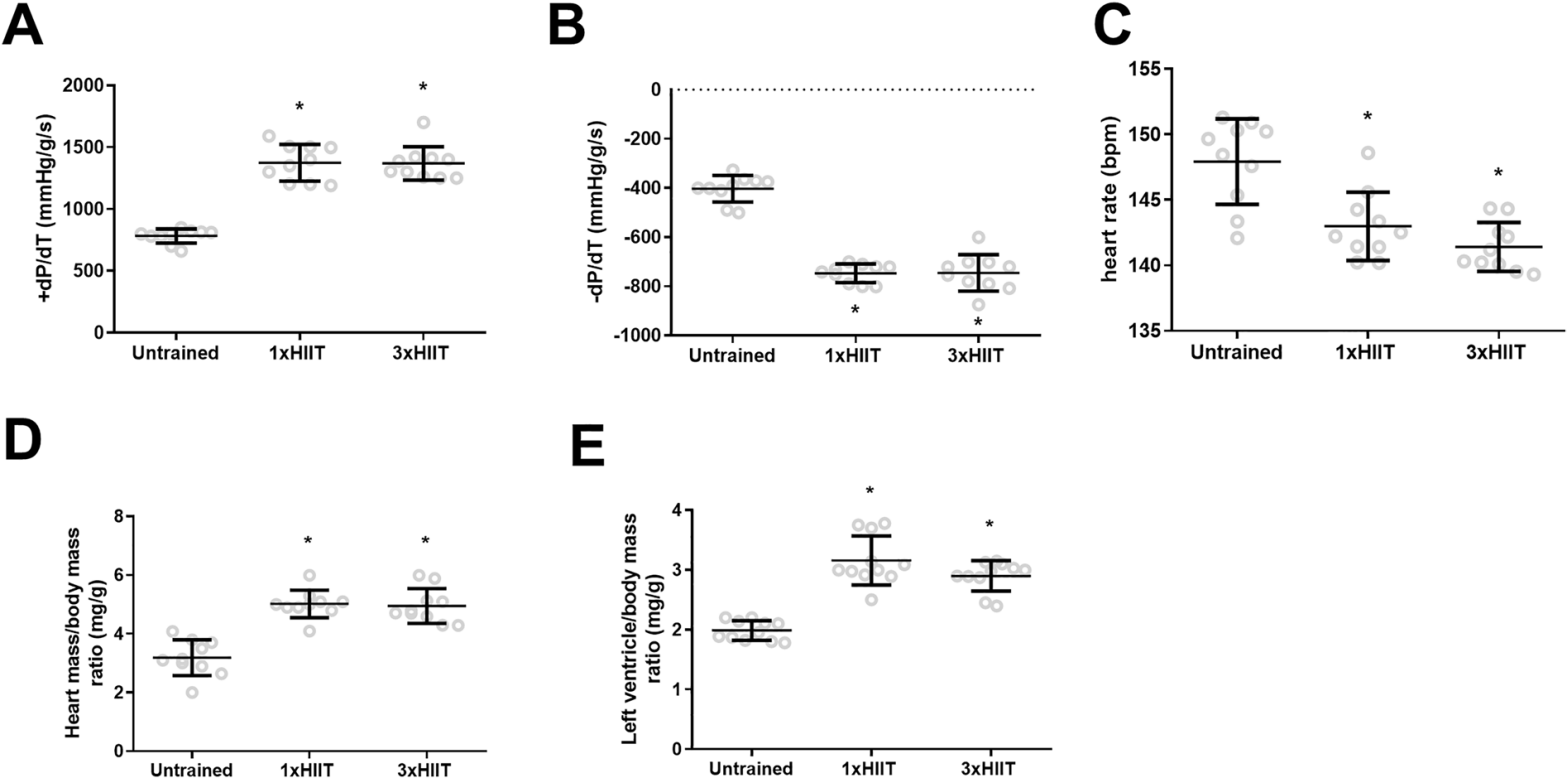
Effects of HIIT performed in single versus three shorter daily sessions on cardiac function and hypertrophy. Index of contractility (+ dT/dt) (**A**). Index of relaxation (− dT/dt) (**B**). Intrinsic heart rate (bpm) (**C**). Heart /body mass ratio (mg/g) (**D**). Left ventricle /body mass ratio (**E**). N = 10/group. Data are presented as mean ± SD. One-way ANOVA followed by the Tukey test. *p < 0.05, HIIT groups versus Untrained Group. 1xHIIT: High-Intensity Interval Training performed in single daily sessions; 3xHIIT: High-Intensity Interval Training performed in three shorter daily sessions.

Figure 5 shows the results of cardiac function and hypertrophy after training. Data obtained from isolated perfused hearts provided direct evidence that both HIIT protocols similarly increased heart function. Both HIIT protocols improved cardiac contractility (1xHIIT vs. Untrained: *p* < 0.0001; 3xHIIT vs. Untrained: *p* < 0.0001; 1xHIIT vs. 3xHIIT: *p* = 0.9941; Fig. 5A) and relaxation (1xHIIT vs. Untrained: *p* < 0.0001; 3xHIIT vs. Untrained: *p* < 0.0001; 1xHIIT vs. 3xHIIT: *p* = 0.9985; Fig. 5B) indexes and reduced intrinsic heart rate (1xHIIT vs. Untrained: *p* = 0.0008; 3xHIIT vs. Untrained: *p* < 0.0001; 1xHIIT vs. 3xHIIT: *p* = 0.3868; Fig. 5C). Both HIIT protocols induced cardiac hypertrophy as shown by the increased heart/body mass ratio (1xHIIT vs. Untrained: *p* < 0.0001; 3xHIIT vs. Untrained: *p* < 0.0001; 1xHIIT vs. 3xHIIT: *p* = 0.9604; Fig. 5D) and left ventricle/body mass ratio (1xHIIT vs. Untrained: *p* < 0.0001; 3xHIIT vs. Untrained: *p* < 0.0001; 1xHIIT vs. 3xHIIT: *p* = 0.1141; Fig. 5E).

**Figure 6:**
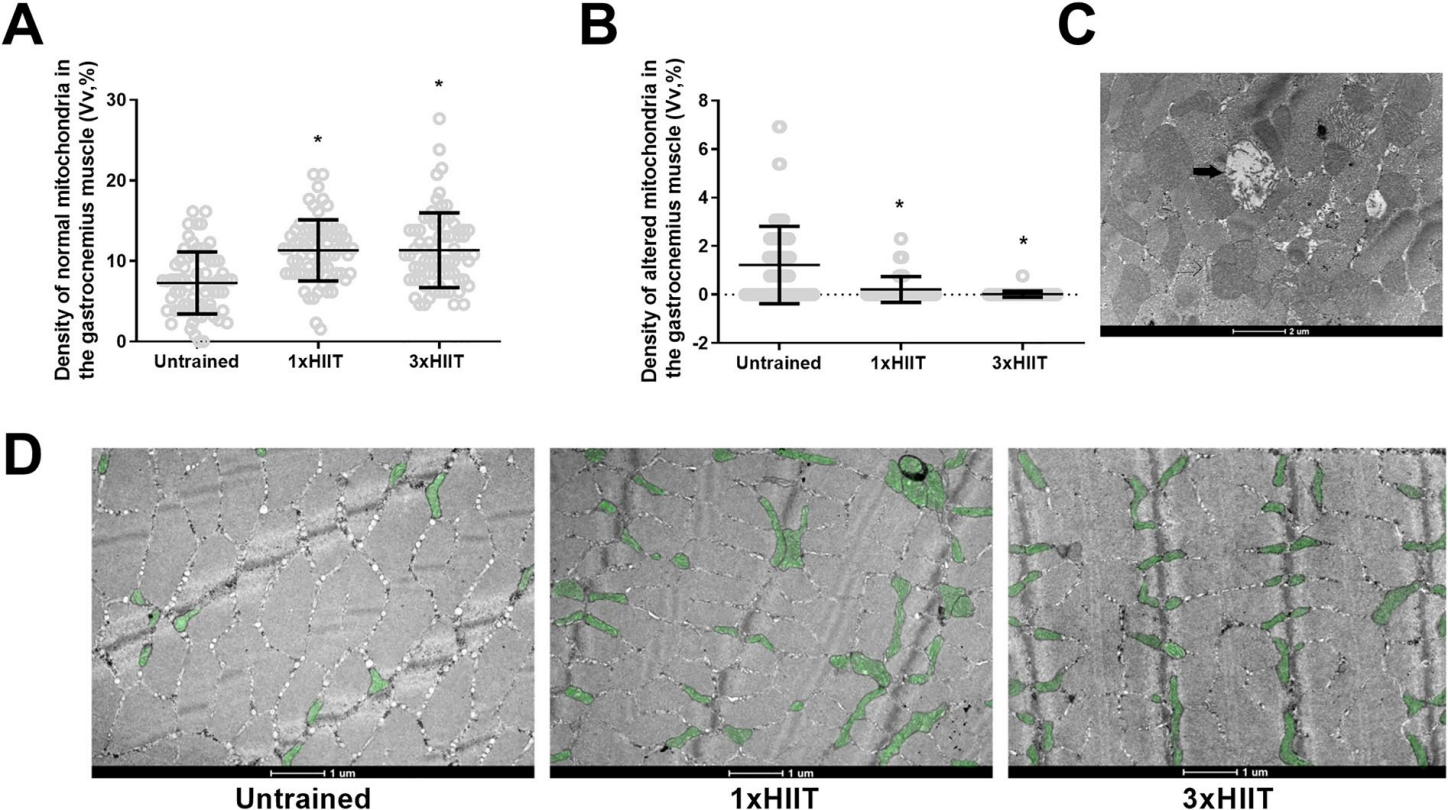
Effects of HIIT performed in single versus three shorter daily sessions on quantification of normal (**A**) and altered (**B**) mitochondria density in gastrocnemius muscle fibers. Transmission electron micrographs of transverse sections of the gastrocnemius muscle (**C**). White arrows show normal mitochondria, and black arrows show altered mitochondria. Representative mitochondria of the gastrocnemius muscle for Untrained, 1xHIIT, and 3xHIIT groups (**D**). Data are presented as mean ± SD. One-way ANOVA followed by the Tukey test. *p < 0.05, HIIT groups versus Untrained Group. 1xHIIT: High-Intensity Interval Training performed in single daily sessions; 3xHIIT: High-Intensity Interval Training performed in three shorter daily sessions.

Figure 6 presents the mitochondrial density analysis of the gastrocnemius muscle. Both HIIT protocols increased the density of normal mitochondria (1xHIIT vs. Untrained: *p* < 0.0001; 3xHIIT vs. Untrained: *p* < 0.0001; 1xHIIT vs. 3xHIIT: *p* = 0.9988; Fig. 6A) and reduced the density of altered mitochondria (1xHIIT vs. Untrained: *p* < 0.0001; 3xHIIT vs. Untrained: *p* < 0.0001; 1xHIIT vs. 3xHIIT: *p* = 0.4620; Fig. 6B). Figure 6C shows a representative cross-sectional image of the gastrocnemius muscle using transmission electron microscopy with white arrows indicating normal mitochondria and black arrows, altered mitochondria. Figure 6D presents a representative image of normal mitochondria (in green) from gastrocnemius muscle.

**Figure 7:**
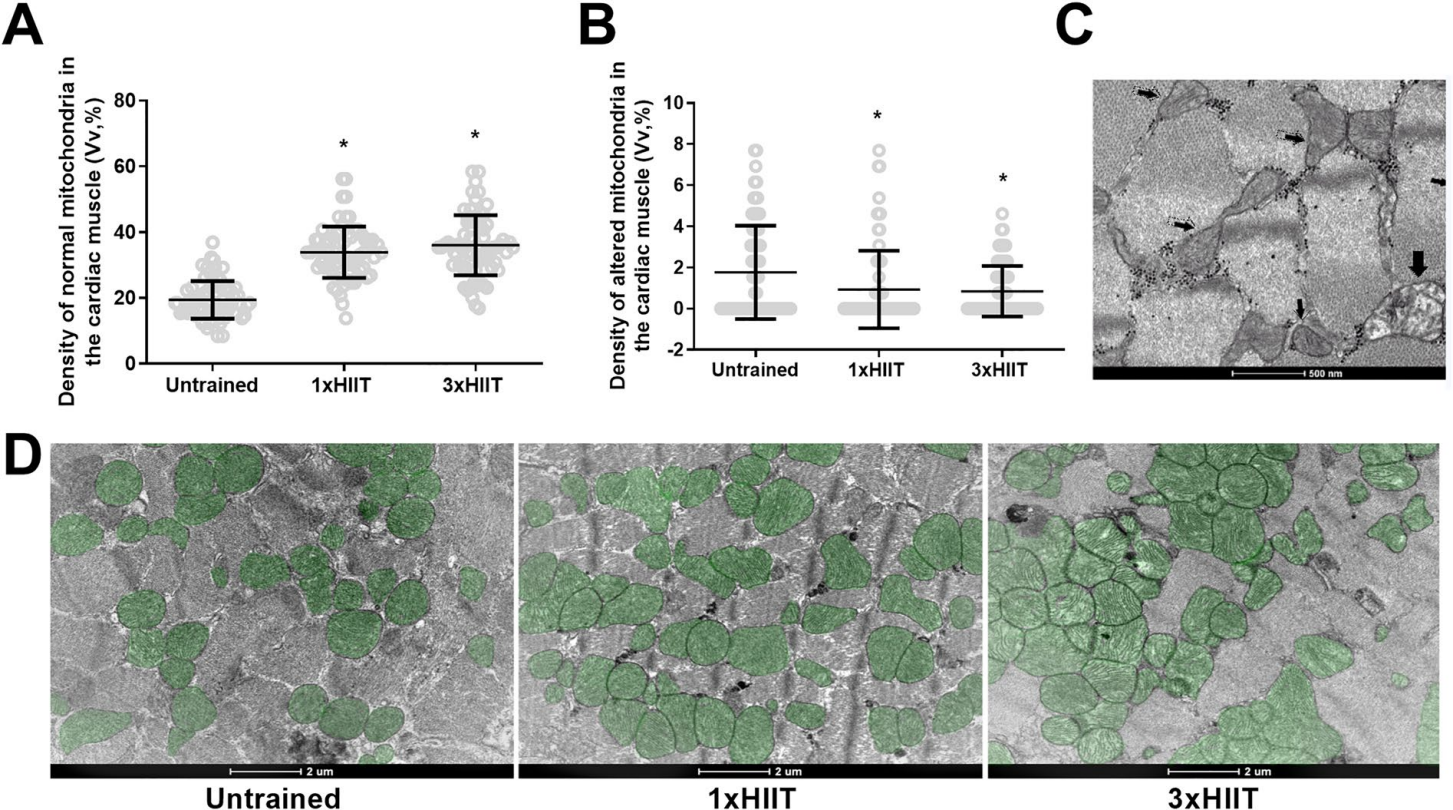
Effects of HIIT performed in single versus three shorter daily sessions on quantification of normal (**A**) and altered (**B**) mitochondria density in cardiac muscle fibers. Transmission electron micrographs of transverse sections of cardiac muscle fibers (**C**). White arrows show normal mitochondria, and black arrows show altered mitochondria. Representative mitochondria of the cardiac muscle fibers for Untrained, 1xHIIT and 3xHIIT groups (**D**). Data are presented as mean ± SD. One-way ANOVA followed by the Tukey test. *p < 0.05, HIIT groups versus Untrained Group. 1xHIIT: High-Intensity Interval Training performed in single daily sessions; 3xHIIT: High-Intensity Interval Training performed in three shorter daily sessions.

Figure 7 presents the mitochondrial density analysis of the cardiac muscle. Similarly to the results of mitochondrial density from gastrocnemius muscle, both HIIT protocols increased the density of normal mitochondria (1xHIIT vs. Untrained: *p* < 0.0001; 3xHIIT vs. Untrained: *p* < 0.0001; 1xHIIT vs. 3xHIIT: *p* = 0.2245; Fig. 7A) and reduced the density of altered mitochondria (1xHIIT vs. Untrained: *p* = 0.0197; 3xHIIT vs. Untrained: *p* = 0.0087; 1xHIIT vs. 3xHIIT: *p* = 0.9586; Fig. 7B) in cardiac muscle. Figure 7C shows a representative cross-sectional image of the myocardium using transmission electron microscopy with white arrows indicating normal mitochondria and black arrows, altered mitochondria. Figure 7D presents a representative image of normal mitochondria (in green) from myocardium.

## Discussion

The present study investigated the effects of HIIT performed in a single or three short daily sessions on cardiometabolic and cellular adaptations of Wistar rats. The main findings were that HIIT performed with sessions of only 5 min three times per day (HIIT “snacks”) was superior to HIIT performed with single daily sessions of 15 min in (i) reducing visceral fat weight and adipocyte size; and (ii) improving insulin resistance. HIIT “snacks” was as effective as HIIT performed with single daily sessions in (i) increasing VO_2_max and resting oxygen consumption; (ii) preventing blood pressure increase; (iii) increasing lean mass and inducing skeletal muscle hypertrophy; (iv) lowering fasting blood glucose levels and increasing glucose tolerance; (v) improving cardiac function and inducing cardiac hypertrophy; and (vi) increasing normal mitochondria density and reducing altered mitochondria density in both gastrocnemius and cardiac muscle fibers. The reasons why training with multiple short bouts of vigorous exercise throughout a day may be similar or even superior to traditional single daily sessions of vigorous exercise are discussed below.

### Blood pressure and heart rate

Both HIIT protocols prevented blood pressure increase. We have previously reported in young Wistar rats that single daily sessions (30–60 min) of MICT prevented blood pressure increase, whereas three short daily bouts (3 × 10–20 min) of MICT did not^10^. In the current study, HIIT performed in single daily sessions (15 min), or three shorter daily sessions (3 × 5 min) prevented blood pressure increase. Thus, interrupting sedentary behavior with regular brief sessions of high-intensity exercise may be more effective in controlling blood pressure than interrupting sedentary behavior with longer sessions of moderate-intensity exercise. Costa-Pereira et al.^11^ found that single or three shorter daily sessions of MICT induced bradycardia. On the other hand, in the current study, single or three shorter daily sessions of HIIT did not induce bradycardia. A recent meta-analysis^19^ of human studies concluded that a HIIT protocol might be superior to MICT in improving endothelial function. Thus, vigorous exercise may improve vascular function without changing sympathetic activity, which may reduce blood pressure independently of heart rate changes. The present study did not compare MICT and HIIT protocols or measure vascular function; however, this is an important area of investigation.

### Cardiac function and hypertrophy

Both HIIT protocols induced similar increases (> 40%) in cardiac contractility/relaxation indexes. These results were similar to our previous study comparing single *versus* multiple daily sessions of MICT in rats^11^. Physiological cardiac hypertrophy induced by exercise training may increase cardiomyocyte contractility and relaxation^20,21^. In this study, both HIIT groups had a 60% increase in cardiac hypertrophy. These findings indicate that cardiac hypertrophy and heart function improvement induced by training may occur even if a longer session of exercise at moderate or vigorous intensity is divided into multiple shorter sessions throughout the day. Future investigations should explore the mechanisms by which single and multiple daily exercise sessions induce the cardiac adaptations of training.

### VO_2_max

After training, both 3 × and 1xHIIT had significantly higher VO_2_max than the Untrained group.One may argue that this difference between trained and untrained groups may be caused by a reduction in VO_2_max in the Untrained rats as a result of their low physical activity levels over 8 weeks of the experimental protocol. However, we can assure that these results occurred as a consequence of the exercise training protocol and not the sedentary behavior of the Untrained group since the Untrained group had a modest reduction in VO_2_max (~ 4%, post- vs. pre-training) whereas the 3xHIIT and 1xHIIT groups had a large increase in VO_2_max (~ 27 and 25%, respectively, post- vs. pre-training).

Studies with humans^8,10^ and animals^11^ have reported similar responses after a period of training with single or multiple short daily sessions of exercise. Little et al.^8^ found in young men that a Sprint Interval Training (SIT) protocol resulted in similar increases in VO2peak after 6 weeks of cycling exercise performed either in a single daily session (3 × 20-s all-out cycling bouts interspersed with 3-min rest) or accumulated in 3 sessions throughout a day (3 × 20-s all-out cycling bouts separated by 1-4 h of rest). Using a MICT protocol for Wistar rats, we also demonstrated similar increases in VO_2_max after 8 weeks of exercise performed either with single daily sessions (30–60 min) or three shorter daily sessions (3 × 10–20 min)^11^. Exercise improves VO_2_max by central and peripheral adaptations. The increase of maximal cardiac output is the most important central mechanism involved with the VO_2_max enhancement^22^. The similar increases in VO_2_max in the 1 × and 3xHIIT animals in the current investigation may, at least in part, be explained by the left ventricular hypertrophy and cardiac function improvement of both exercised groups. In addition, the increase of muscular mitochondrial density is the most important peripheral adaptation involved with VO_2_max increase.

### Skeletal and cardiac muscle mitochondrial density

The current study revealed that cardiac and skeletal muscles of 1 × and 3 × HIIT rats had similar increases in the density of normal mitochondria and decreases in the density of altered mitochondria. Recent studies have demonstrated that a HIIT protocol is superior to a MICT protocol in increasing Peroxisome proliferator-activated receptor-gamma coactivator (PGC)-1alpha expression in the skeletal muscle^23^ and heart^24^ of rodents. Although PGC-1alpha is recognized as a key intracellular protein of mitochondrial biogenesis activation^25^, an increase in its expression does not necessarily induce an increase in mitochondrial biogenesis. Studies with PGC-1-deficient mice revealed that despite these mice presenting lower mitochondrial genes of oxidative phosphorylation in the soleus and cardiac muscles, the mitochondrial density in these muscles is not different between PGC-1-deficient and wild-type mice^26,27^. We highlight the use of transmission electron microscopy in the current study, which is considered the "gold standard" method to measure mitochondria content, size, and shape^28^. This is the first study showing that 8 weeks of a 15-min HIIT protocol increases the density of normal mitochondria and decreases the density of altered mitochondria in cardiac and gastrocnemius muscles of rats. If multiple bouts of high-intensity exercise are performed throughout the day, one may argue that metabolic stress may not reach levels that induce mitochondrial adaptations. However, notably, these responses occurred even when the HIIT protocol was broken up into three shorter 5-min sessions over the course of a day. These results corroborate with the studies of Maclnnis et al.^29^, which demonstrated that increases in exercise intensity are more effective than increases in exercise duration for raising muscle mitochondria content.

### Body composition

Both 1 × and 3xHIIT protocols increased lean mass and induced skeletal muscle hypertrophy. However, the 3xHIIT protocol was superior to the 1xHIIT protocol in decreasing visceral fat content and adipocyte size. The superiority of 3xHIIT in reducing visceral fat mass and adipocyte area occurred independently of energy intake. These results are in agreement with our previous study in which 8 weeks of MICT performed in three shorter daily sessions was superior to single daily sessions in reducing visceral fat and adipocyte size independently of energy intake^11^. In the current study and in the study by Costa-Pereira et al., rats exercised at the same intensity and total duration; thus, the difference between rats that exercised with single versus multiple daily sessions was the exercise frequency. Sene Fiorese et al.^30^ hypothesized that multiple daily exercise sessions might result in higher energy expenditure than single daily sessions due to the larger availability of fatty acids for oxidation during each one of the multiple daily exercise sessions. Murphy et al.^31^ further hypothesized that the sum of the small increases in metabolism after each of multiple daily exercise sessions (known as Excessive Post-exercise Oxygen Consumption- EPOC) may be greater than the EPOC of a longer single daily session of exercise. All these hypotheses warrant future investigation.

### Glucose homeostasis

Both 1 × and 3 × HIIT protocols reduced fasting glucose levels and enhanced glucose tolerance. Nevertheless, only the 3xHIIT protocol enhanced insulin tolerance. These results indicate a superior effect of multiple versus single daily sessions of HIIT on insulin sensitivity. Although the sedentary control rats were not insulin-resistance, the possibility that multiple daily vigorous exercise sessions are more effective than single ones on glucose homeostasis control seems an interesting strategy for diabetes prevention. We previously found that training with multiple daily sessions of MICT was as effective as single daily sessions in improving the glucose tolerance of young rats; however, insulin tolerance was not evaluated in that study^11^. The physiological mechanisms responsible for improvements in glycemic control and insulin sensitivity following a period of HIIT protocol are unclear. The great reduction of muscle glycogen induced by HIIT is supposed to activate intracellular pathways involved with insulin sensitivity in skeletal muscle^32^. Dela et al. demonstrated that a HIIT session improved skeletal muscle insulin sensitivity of individuals with type 2^32^. The present study did not address the mechanisms responsible for the greater improvement in insulin tolerance following 3xHIIT compared to 1xHIIT protocol, although this is an important area of investigation in future studies.

### Limitations and strengths

A complete characterization of mechanisms underlying the effects of the 1 × *versus* 3xHIIT protocols was beyond the scope of the present study.

Moreover, the current study's findings were obtained with rats and cannot be directly transferred to humans. Thus, the confirmation in human studies of the superiority of HIIT “snacks” to traditional single daily HIIT sessions for cardiometabolic health will have practical implications, as this strategy may be a feasible approach to increasing exercise adherence.

However, we highlight some advantages of using rats in the present study: (i) the use of lab animals to study the effects of training with very low volume exercise sessions overcomes some methodological pitfalls of studies in humans, such as the fine control of energy intake/food quality, temperature/humidity environment, and several other variables that are difficult to control accurately in human free-living sceneries. (ii) the possibility of investigating training adaptations that are not possible with humans, such as the direct evaluation of cardiac function in isolated hearts or the direct evaluation of normal and altered mitochondria densities in the heart. (iii) Both HIIT protocols used in this study may be applied in future human clinical trials.

## Conclusion

Data from the present study reveal that multiple daily short sessions of HIIT (HIIT “snacks”) induce superior training adaptations to single daily HIIT sessions, such as reduction of visceral fat weight and adipocyte size and improvement of insulin tolerance. Thus, this study provides the first evidence that HIIT “snacks” might be superior to traditional single daily HIIT sessions in promoting cardiometabolic health, especially in preventing obesity and insulin resistance in rats.

## Data Availability

The datasets used and analyzed during the current study are available from the corresponding author on request.
